
JOURNAL INFORMATION
==============================
NLM Title Abbreviation: Behav Neurol
Journal ID: Behav Neurol
NLM Journal ID: 8914585
Journal ID: BN

Behavioural Neurology

ISSN: 0953-4180
EISSN: 1875-8584
Publisher: Wiley

ARTICLE INFORMATION
==============================
PMCID: PMC5469969
PMID: 17726249
DOI: 10.1155/2007/274156
Article ID: 274156
Article version: 1
Subjects: Clinical Note

De Clerambault’s Syndrome (Erotomania) As a Presenting Feature of Fronto-Temporal Dementia and Motor Neurone Disease (FTD-MND)

Olojugba Charles 1 3 *
de Silva Rajith 2
Kartsounis Luke D. 2
Royan Lindsay 1
Carter Janet 1 4
1 The Petersfield Centre Essex RM3 9PB UK
2 Department of Neurology Essex Centre for Neurological Sciences Queen’s Hospital Romford RM7 0AG UK
3 St. Pancras Hospital London NW1 OPE UK
4 Department of Mental Health Sciences University College London London W1N 8EY UK
*Charles Olojugba: magdac@supanet.com
Print publication date: 2007
Electronic publication date: 2007 Aug 22
Volume: 18
Issue: 3
First page: 193
Last page: 195
Received 2007 Aug 14; Accepted 2007 Aug 14
Copyright: Copyright © 2007 Hindawi Publishing Corporation and the authors.
Copyright year: 2007
License: This is an open access article distributed under the Creative Commons Attribution License, which permits unrestricted use, distribution, and reproduction in any medium, provided the original work is properly cited.
License URL: https://creativecommons.org/licenses/by/3.0/

Keywords: De Clerambault’s syndrome, erotomania, fronto-temporal dementia, motor neurone disease

==============================
Behavioural Neurology 18 (2007) 193–195
IOS Press

Clinical Note

### De Clerambault's syndrome (erotomania) as a presenting feature of fronto-temporal dementia and motor neurone disease (FTD-MND)

Charles Olojugba[a,c,*], Rajith de Silva[b], Luke D Kartsounis[b], Lindsay Royan[a] and Janet Carter[a,d]
[a]*The Petersfield Centre, Essex RM3 9PB, UK*
[b]*Department of Neurology, Essex Centre for Neurological Sciences, Queen's Hospital, Romford RM7 0AG, UK*
[c]*St. Pancras Hospital, London NW1 OPE, UK*
[d]*Department of Mental Health Sciences, University College London, London W1N 8EY, UK*

Keywords: De Clerambault's syndrome, erotomania, fronto-temporal dementia, motor neurone disease

### 1. Introduction

Motor neurone disease (MND) is characterized by the progressive degeneration of lower and upper motor neurones in the spinal cord, brainstem and motor cortex of the brain, leading to a variable pattern of motor dysfunction. Typical symptoms include muscle atrophy, limb weakness, bulbar palsy and respiratory failure. Fronto-Temporal Dementia (FTD) is a common neurodegenerative disorder associated with progressive atrophy of the frontal and temporal lobes of the brain, resulting in changes in personality and behaviour and/or speech and language dysfunction. Fifty per cent of patients with MND exhibit frontal executive deficits during the course of their illness, representing in most instances the co-occurrence of FTD. Previous descriptions of FTD indicate that psychotic symptoms (including hallucinations and delusions) are rare and specifically cases of erotomania in FTD have not to our knowledge been previously described [1].

Erotomania or De Clerambault's syndrome is a delusional disorder typically presenting in middle-aged females in association with psychiatric illness, including Schizophrenia and Bipolar Affective Disorder [1]. It is characterised by the delusional belief that another person (the object), who is socially unobtainable because of unbridgeable gaps in social and financial statuses, passionately loves the patient. In this paper, a case of FTD-MND in an elderly woman presenting with erotomania is reported.

### 2. Case report

The patient was a 65-year-old divorcee living alone in her own flat. Over a six-month period she had become increasingly preoccupied with the late celebrity Dick Haymes (DH), who was one of the most popular

*Address for correspondence: Dr. Charles Olukunle Olojugba, MBBS BSc MRCpsych DGM-RCP, Specialist registrar in old age psychiatry, St. Pancras Hospital, St. Pancras Way, London NW1 OPE, UK. Tel.: +44 207 530 4400; Fax: +44 207 540 4211; E-mail: magdac@supanet.com.

ISSN 0953-4180/07/$17.00 © 2007 – IOS Press and the authors. All rights reserved

American male vocalists of the 1930s–40s known for his love ballads and marriage to the Hollywood actress Rita Hayworth. She believed that DH was in love with her and was living next door to her. She had purchased multiple copies of CDs and DVDs featuring DH, and had even waited for hours outside her flat anticipating DH's arrival. She had shown signs of aggression by slapping her daughter when challenged about her behaviour. In addition she was not eating and sleeping properly, and had been wandering the streets at odd times.

She had no previous psychiatric history. Her family history revealed panic attacks, agoraphobia and auditory hallucinations in her mother and alcoholism in her father. Her medical history included a previous transient ischaemic attack and non-arteritic anterior ischaemic left optic neuropathy. She smoked 40 cigarettes a day until 5 years previously and had not abused alcohol. She had been married for 18 years before becoming divorced, and had two children. On initial examination, she was mildly dysarthric but no other neurological abnormalities were noted. Her mood was elated and at times she was disinhibited, for example laughing inappropriately. She had a fixed belief that DH was in love with her and she with him, stating "I love him to bits". She was preoccupied with this theme and it was difficult to distract her. Despite being told that DH was dead she insisted that he was alive. There was no evidence of hallucinosis in any modality and her mini mental state examination score was 29/30. More detailed cognitive testing indicated impaired abstracting ability, and planning and organisational difficulties. MRI of the brain showed chronic small vessel ischaemia and atrophy affecting the anterior temporal lobes. An HMPAO-SPECT scan demonstrated hypoperfusion especially of the left fronto-temporal region (Fig. 1). She was started on olanzapine 2.5 mg to which she responded well and her delusions abated. Subsequently her speech deteriorated rapidly, becoming more dysarthric and dysphonic. She had difficulty swallowing food, and she developed wasting of the small muscles in her hands, resulting in loss of function. She developed progressive weakness of her legs, and had weight loss. The patient was referred urgently for a neurological opinion, eighteen months after her initial presentation. At that point, she was anarthric, with restricted soft palate movement and fasciculations in her tongue. A left-sided palmomental and pouting responses were present. In her upper limbs she had profound small hand muscle wasting bilaterally with clawing. Profuse fasciculations were present in the upper and lower limbs. Limb reflexes were present, and plantar responses were flexor. A speech and language therapy assessment including video fluoroscopy demonstrated oral stage dysphagia characterised by weak tongue and reduced soft palate movements. Electrophysiology confirmed widespread acute and chronic partial denervation, suggestive of MND.

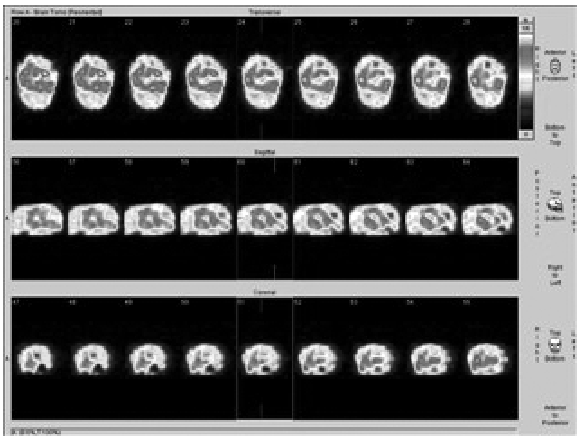

Fig. 1. HMPAO-SPECT scan demonstrating hypoperfusion of the fronto-temporal region.

Neuropsychological examination revealed that the patient had marked difficulty in sustaining concentration over time precluding comprehensive assessment. She became "stimulus bound" with any change in the environment, and showed motor perseveration and poor self-monitoring. She was impulsive in her responses and exhibited utilisation behaviour, attempting to use any object placed in her vicinity. As a result, testing could proceed only by the examiner physically restraining her from making impulsive responses. On Matrix Reasoning, a set of nonverbal reasoning problems of the Wechsler Adult Intelligence Scale [2], she scored at a borderline level. On the Test for Reception of Grammar [4], she performed creditably at a single word level. She appeared able to comprehend only very simple sentences. She was able to match a few printed single words to corresponding pictures. She showed good recognition of common objects. On the Object Decision Test of visual perception [5] she performed within the normal range for her age group (15/20). She was unable to complete the Weigl Colour From Sorting Test, a relatively undemanding test of nonverbal abstract reasoning. These observations suggested that her posterior cortical cognitive functions were probably well preserved. By contrast, she presented with profound executive deficits, with environmental dependency, impulsivity, poor self-monitoring and elements of utilisation behaviour, indicative of marked frontal lobe dysfunction. A diagnosis of MND with FTD was made, and she was started on Riluzole.

### 3. Discussion

Erotomania in the elderly is a rare presenting symptom. Initial examination of our patient revealed executive deficits and mild dysarthria in addition to the delusional disorder. Thus, she showed cognitive and physical evidence of a neurological disorder at presentation, as well as a psychiatric syndrome. The neuroimaging data, the presence of frontal executive dysfunction, despite an initial normal MMSE score, and mood disturbance supported a diagnosis of fronto-temporal dementia, according to the Lund-Manchester diagnostic criteria [7]. The development of progressive bulbar signs prompted a second neurological examination, which identified wasting of the small hand muscles and gait impairment – leading to a diagnosis of MND. This highlights the importance of repeating the neurological evaluation of elderly patients who present with psychiatric syndromes that worsen despite treatment, especially when new neurological complaints develop.

In FTD-MND psychotic symptoms are relatively rare [6]. In a previous study of three cases of psychosis in FTD-MND, psychotic symptoms did not predate the onset of motor signs of MND. However, delusions when present were always of a persecutory content. Additionally in the cases studied all the patients experienced hallucinations as part of their psychosis. The early involvement of the temporal lobes in the degenerative process was suggested to have been a possible cause of hallucinations. No explanation for the occurrence of delusions was offered [9]. In contrast, the delusions in the patient reported here were erotomanic in nature and unassociated with hallucinations. The explanations for the observed differences in these cases are unknown, but premorbid factors such as personality may play a role.

In Alzheimer's disease where delusions are reported in 30%–40% of cases, PET neuroimaging has demonstrated a significant correlation between hypometabolism in the right prefrontal cortex and delusional thought [3]. However, a SPECT analysis in patients with FTD failed to show any association between frontotemporal hypoperfusion and psychotic symptoms [8], further highlighting the uncertainty surrounding the origin of delusions in FTD-MND.

### 4. Conclusion

FTD-MND can present with delusional disorder. The neuronal mechanisms underlying this type of presentation are presently unclear. More research is needed to evaluate the clinical course of such patients.

*Declaration of interest: none.*

*The authors acknowledge the contributions of Dr. Sanjiv Chawda (Neuroradiology), Dr. Ranjan Gunasekera (HMPAO-SPECT data), Dr. Santiago Catania (Clinical Neurophysiology), and Ms. Melanie Whitehead (swallowing assessments).*

### References

[1] B. Martin and S. Stefan, Erotomania variants in dementia, *J Geriatr Psychiatry and Neurol* **16** ( 2003), 232–234.
[2] D. Wechsler, *Wechsler Adult Intelligence Scale (WAIS-III)*, London, The Psychological Corporation, 1997.
[3] D.L. Sultzer and C.V. Brown, Delusional thoughts and regional frontal/temporal cortex metabolism in Alzheimer's disease, *Am J Psychiatry* **161** (2004), 764.
[4] D.V.M. Bishop, *The Test for Reception of Grammar (TROG)*, Cambridge, Medical Research Council, 1992.
[5] E.K. Warrington and M. James, *The Visual Object and Space Perception Battery*, Bury St Edmunds, Thames Valley Test Co, 1991.
[6] J. Hodges, R. Davies and J. Xuereb, Clinicopathological correlates in frontotemporal dementia, *Ann Neurol* **108** (2004), 515–523.
[7] Lund and Manchester Groups Clinical and neuropathological criteria for frontotemporal dementia, *J Neurol Neurosurg Psychiatry* **57** (1994), 416–418.
[8] M.F. Mendez and A. McMurtray, Functional neuroimaging and presenting psychiatric features in frontotemporal dementia, *J Neurol Neurosurg Psychiatry* **77** (2006), 4–7.
[9] R. Nitrini and S. Rosemberg, Psychotic symptoms in dementia associated with motor neurone disease: a pathological hypothesis, *J Neuropsych Clin Neurosci* **10** (1998), 456–458.
